# Clinical Outcomes of Benzodiazepine Prescribing for People Receiving Opioid Agonist Treatment: A Systematic Review of the Evidence

**DOI:** 10.3390/pharmacy12050152

**Published:** 2024-10-04

**Authors:** Catriona Matheson, Chris Vucic, Josh Dumbrell, Roy Robertson, Trina Ritchie, Clare Duncan, Karthigayan Kessavalou, Caroline Woolston, Joe Schofield

**Affiliations:** 1 Faculty of Applied Social Science, University of Stirling, Stirling FK9 4LA, UKj.l.dumbrell@stir.ac.uk (J.D.); 2 The Usher Institute, BioQuarter, Little France, Edinburgh EH16 4UX, UK; roy.robertson@ed.ac.uk; 3 Glasgow Alcohol and Drug Recovery Services, NHS Greater Glasgow and Clyde, Glasgow G1 1DH, UK; trina.ritchie@nhs.scot; 4 NHS Ayrshire and Arran, 35 Lister Street, Crosshouse Hospital, Kilmarnock KA2 0BE, UK; clare.duncan@aapct.scot.nhs.uk (C.D.); karthigayan.kessavalou@aapct.scot.nhs.uk (K.K.); caroline.woolston@aapct.scot.nhs.uk (C.W.); 5 School of Medicine, University of St Andrews, St Andrews KY16 9TF, UK; js534@st-andrews.ac.uk

**Keywords:** benzodiazepine prescription, opioid replacement treatment, opioid agonist treatment, illicit drug use, street benzodiazepines, drug overdose, mortality, clinical outcomes, addiction, clinical decision-making

## Abstract

Many countries are experiencing an increased use of unregulated benzodiazepines in combination with opioids and other drugs, which contributes to drug-related harm. This descriptive review identifies and synthesises the outcomes of studies co-prescribing benzodiazepines and opioids. A systematic review was undertaken in Medline, CINAHL, PsychInfo, Embase, and the Cochrane databases covering publications from 1 January 1991 to 18 November 2021. Inclusion criteria were peer reviewed, English language studies of adults prescribed opioid agonist treatment (OAT) and a concurrent benzodiazepine, and reporting outcome data. Of the 4370 titles screened, 18 papers were included. The main outcomes identified covered all-cause mortality (ACM) (n = 5); overdose death (n = 3); retention in treatment (n = 7); and hospitalisation/emergency department encounters (n = 2). Other outcomes included QTc interval, cognitive function, illicit drug use, and mental health. The prescription of benzodiazepines alongside OAT increased the ACM by 75–90%, while evidence on overdose death was less robust but indicative of increased risk (40–334%). There was an indicative positive effect on treatment retention, with increased retention in those prescribed a benzodiazepine with OAT compared to those not prescribed or taking non-prescribed benzodiazepines. In conclusion, methodologically robust epidemiological studies found increased ACM and overdose death but possibly improved retention. However confounders (e.g., psychiatric comorbidity) exist, so a trial is recommended.

## 1. Introduction

Benzodiazepines have been used across the world for decades. Many parts of the world are now experiencing an increased use of unregulated benzodiazepines, often in combination with other substances such as opioids. This wave of benzodiazepines (also referred to as designer benzodiazepines) follows a trend in new psychoactive substance use and contributes to drug-related harm [1]. Unregulated benzodiazepines include those that can be prescribed but are diverted from prescription sources, or counterfeit benzodiazepines. In addition, there are new benzodiazepines that have been synthesised to have benzodiazepine-like features, but are not regulated by the current legislation. Whether prescribed or through an unregulated supply, benzodiazepines, as sedative drugs, pose an additional risk of overdose due to the potentiation of respiratory depression when used concurrently with opioids. In Scotland, benzodiazepines were implicated in 601 (57%) drug-related deaths (DRD) in 2022 [2]. In England. whilst less than in Scotland, the levels of benzodiazepines implicated in deaths are rising [3]. These deaths are generally in combination with other drugs, most commonly opioids. Across the UK, and in Scotland in particular, these substances are predominately substances not licensed for prescription such as etizolam (a thenodiazepine) and a range of alprazolam derivatives [4]. New variations continue to appear. This pattern of increased benzodiazepine use/misuse is also becoming evident in, for example, Northern Ireland [5] and the USA [6].

The prevalence of unregulated benzodiazepine use is known to be higher among people with other substance-use disorders, especially problematic opioid and/or alcohol dependence [7]. A systematic review identified a high prevalence (typically > 40%) among people on opioid agonist treatment (OAT) [8].

Current clinical guidance in the UK only supports maintenance prescriptions of benzodiazepines in exceptional cases [9], because the evidence of patient safety and other outcomes was still sparse and conflicting at the time the guidelines were developed. A 2018 Cochrane review of methods to discontinue benzodiazepines concluded that “it is not possible to draw firm conclusions regarding pharmacological interventions to facilitate benzodiazepine discontinuation in chronic benzodiazepine users” [10]. The poor evidence base for the co-prescribing of benzodiazepine in people on OAT who face increased risk of respiratory depression has been noted [11]. However, the published evidence of benzodiazepine prescribing among people on OAT has expanded since these reviews and guidelines, and there is descriptive evidence from across the world of the concurrent prescribing of benzodiazepines and OAT.

Concurrent prescriptions of benzodiazepines and opioid agonists, such as methadone or buprenorphine used for OAT, are prevalent across several countries. A study of U.S. military veteran prescription services in 2012 found up to 178 out of 1091 (16%) veterans received concurrent buprenorphine and benzodiazepine prescriptions [12]. Of these, 75 (42%) had overlapping prescribing periods in excess of 90 days. Similarly, a U.S. study in 2010 found that up to 38.5% of veterans receiving OAT were also prescribed a benzodiazepine [13]. Another study of eight U.S. states in 2013 found that out of 190,907 patients, around 12.5% received both a buprenorphine and benzodiazepine prescription [14]. In Norway, 50% of 10,371 OAT patients from 2013 to 2017 were dispensed benzodiazepines or z-hypnotics at least once [15].

This systematic review was conducted to inform clinical decision-making around the risk of co-prescribing a benzodiazepine and OAT. This is of particular importance in countries like Scotland, where unregulated benzodiazepines are considered to be driving morbidity and mortality among people who use drugs [4], and clinicians are assessing risks and benefits.

## 2. Materials and Methods

### 2.1. Aim

The aim of the review was to describe international evidence on the clinical outcomes of the prescribing of benzodiazepines among people receiving treatment for opiate/opioid addiction (the term opioid will be used hereafter for simplicity). The review was registered on PROSPERO (CRD42021282474).

### 2.2. Inclusion and Exclusion Criteria

The review was conducted and documented in line with the PRISMA-P checklist [16]. The following types of studies were eligible for inclusion: observational/interventional epidemiological studies; trials and other comparative clinical studies; service evaluations; systematic/scientific integrity reviews; and meta-analyses. Studies were included if their participants included people aged ≥18 years who were prescribed OAT and a benzodiazepine, and if the outcome data were reported. Outcomes of interest included: death (all-cause mortality (ACM) and drug-related death); emergency department encounters or hospitalisation (all-cause and drug-related); and retention in drug treatment.

### 2.3. Search Strategy and Data Extraction

A search strategy was developed to identify publications containing combinations of keywords and adapted for the Medline, CINAHL, PsychInfo, Embase, and Cochrane databases, which were searched on 18/11/21. The search terms are provided in the Supplementary Materials.

The results were combined, and the duplicates removed (JS). Titles were screened, followed by an abstract screen (JS, CM). Full papers were reviewed against the inclusion criteria with a discussion where there was some uncertainty (CM or CV). Detailed data extraction was then undertaken by a second reviewer (TR, RR, JD, KK, CD, CW), with a further reviewer (CM, RR) reviewing any papers where there was uncertainty.

Descriptive information, sample characteristics, and outcomes data were extracted for each paper using a standard data extraction form. Descriptive information included study design, country, and year. Sample characteristics included sample size and demographics.

### 2.4. Analysis

A meta-analysis was considered inappropriate due to the heterogeneity of the methods and outcomes in the included studies. A descriptive synthesis was undertaken in which studies were grouped by outcome.

### 2.5. Quality Assessment Review

The Mixed Methods Appraisal Tool (MMAT), a critical appraisal tool designed for use in systematic reviews that include studies with a range of methodologies, was selected for this review [17]. Due to the subjective nature of critical appraisal, each study was reviewed against the MMAT criteria by two reviewers (CM and JD or CV).

## 3. Results

**Figure 1:**
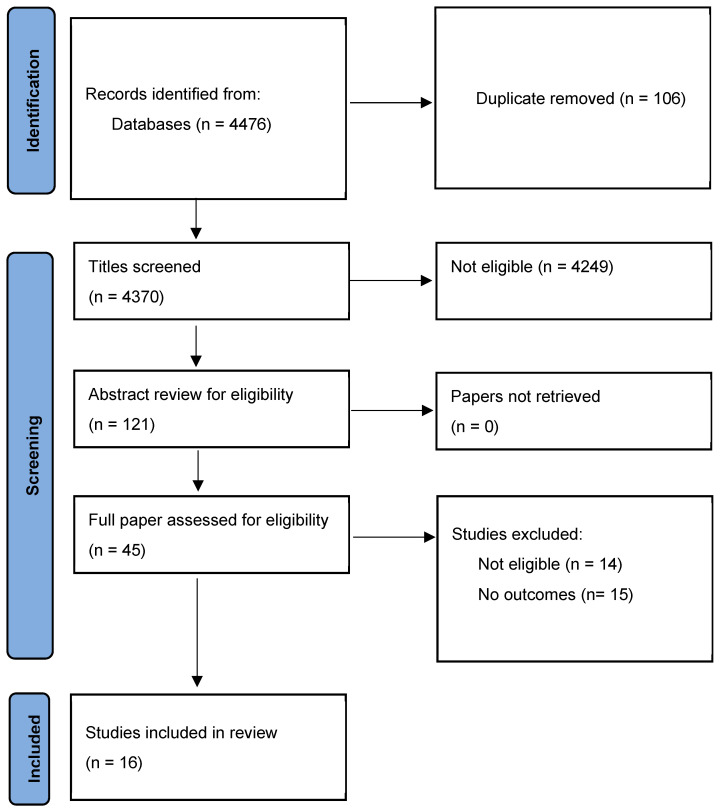
PRISMA diagram of study screening and review.

The search is described in the PRISMA chart (Figure 1). After screening and review, eighteen papers were identified for inclusion [18,19,20,21,22,23,24,25,26,27,28,29,30,31,32,33] (see Figure 1).

### 3.1. Quality Assessment

The quality of studies was variable, and quality assessment is referred to when individual study findings are presented. Summarised results of the MMAT screening are presented in Table 1 according to study type.

### 3.2. Study and Sample Characteristics

Summaries of the included studies are presented in Table 2. The review included reports on epidemiological (cohort/case controlled) studies and controlled trials whose participants were patients recruited from national cohorts (via administrative data), individual clinics, or groups of clinics. The included studies covered a range of countries including Europe, Scandinavia, North America, and Israel. Participating patients included males and females, typically in an age range of 29–39 years. All were receiving OAT at the time of the study, and this may have been a new or first treatment episode, however, one study specified inclusion as the first OAT treatment. For simplicity, BZD is used for benzodiazepine.

**Table 1:** Quality assessment screening using the Mixed Methods Appraisal Tool.

**Randomised Controlled Trials**								
**First Author,** **Year**	**Are the Research Questions Clear?**	**Do the Collected Data Address the Research Questions?**	**Is Randomisation Appropriately Performed?**	**Are the Groups Comparable at Baseline?**	**Are There Complete Outcome Data?**	**Are Outcome Assessors Blinded to the Intervention Provided?**	**Did the Participants Adhere to the Assigned Intervention?**	**Comments**
Eiroa-Orosa, 2010 [23]	Yes	Yes	Cannot tell	Yes	Can’t tell	Can’t tell	Yes	Does not describe how the randomisation schedule was generated.
**Quantitative non-randomised**								
**First author,** **Year**	**Are the Research Questions Clear?**	**Do the Collected Data Allow the Research Questions?**	**Are the Participants Representative of the Target Population?**	**Are Measurements Appropriate Regarding Both the Outcome and Intervention (or Exposure)?**	**Are There Complete Outcome Data?**	**Are the Confounders Accounted for in the Design and Analysis?**	**During the Study Period, Was the Intervention Administered (or Exposure Occurred) as Intended?**	**Comments**
Abrahamsson, 2017 [18]	Yes	Yes	Yes	Yes	Yes	Cannot tell—see comment	Yes	Individuals were excluded if they had a prescription for pain, but 22% of the sample were prescribed pregabalin.
Durand, 2021 [21]	Yes	Yes	Yes	Yes	Yes	Cannot tell—see comment	Yes	Possible selection bias, as they excluded all in continuous MMT prior to study start date.
Eibl, 2019 [22]	Yes	Yes	Yes	Yes	Yes	Cannot tell	Yes	
MacLeod, 2019 [24]	Yes	Yes	Yes	Yes	Yes	Cannot tell	Yes	
Maremmani, 2014 [25]	Yes	Yes	Yes	Yes	Yes—per protocol	Cannot tell	Yes	Clinical setting, observational study, unable to follow up patients who dropped out.
Park, 2020 [28]	Yes	Yes	Yes	Yes	Yes	No	Yes	-
Rapeli, 2009 [29]	Yes	Yes	Yes	Yes	Yes	Yes	Yes	Did not compare OAT vs. OAT and BZD.
Schuman-Olivier, 2013 [30]	Yes	Yes	Yes	Yes	Yes	No	Yes	
Sharma, 2020 [31]	Yes	Yes	Cannot tell	Yes	Yes	Yes	Yes	A broader population, not just OAT patients.
Weizman, 2003 [32]	Yes	Yes	No	Yes	No	Yes	Yes	
Xu, 2021 [33]	Yes	Yes	Yes	Yes	Yes	Yes	Yes	Case–controlled.
**Quantitative descriptive**								
**First author,** **Year**	**Are the Research Questions Clear?**	**Do the Collected Data Allow the Research Questions?**	**Is the Sampling Strategy Relevant to Address the Research Question?**	**Is the Sample Representative of the Target Population?**	**Are the Measurements Appropriate?**	**Is the Risk of Nonresponse Bias Low?**	**Is the Statistical Analysis Appropriate to Answer the Research Question?**	**Comments**
Bakker and Streel, 2017 [19]	Yes	Yes	Yes	Cannot tell (as it is a practice population)	Yes	Yes	Yes	Studied all OAT patients in one practice, not representative of wider population. Inferential tests limited to retention analysis (*t*-tests).
Best, 2002 [20]	Cannot tell	Cannot tell	Cannot tell	Cannot tell	Yes	No	Cannot tell (see comment)	Attrition bias possible (24%). Does not explicitly state which analyses were used.12 3.
Mijatović, 2013 [26]	Yes	Cannot tell	Cannot tell	Cannot tell	Cannot tell	Yes	Yes	Does not mention sampling method. Patients in one Serbian drug treatment centre; excluded patients with severe physical disease, mental disorders and polysubstance dependence. Unclear how side-effects (besides QTc) were measured.
Mijatović, 2017 [27]	Yes	Yes	Cannot tell	Cannot tell	Yes	No	Yes	30 patients enrolled in study; 17 patients present six months after starting MMT.

**Table 2:** Summary of included studies.

First Author, Year	Country of Study	Aim	Design	Outcome Measures	Sample Size	Demographics	Summary of Findings
Abrahamsson, 2017 [18]	Sweden	Assess whether prescription of sedatives may be associated with mortality in patients in opioid maintenance treatment.	Open cohort	Mortality.	4501	26.2% female. Median (IQR) age at baseline 34.4 (28.7–42.1) yrs.	Association between z-drug and pregabalin prescriptions and overdose deaths in subjects in OAT. For BZD prescriptions (all prescribable BZD included) the association with overdose death was unclear, whereas the association with non-overdose death was significant.
Bakker, 2017 [19]	UK	Describe co-prescribing of BZD and OST, investigate links with retention in care and mortality.	Case note review	Treatment retention, mortality.	278	30.6% female. 81% White, 10% Arab, 4% Asian, 4% Black.	Treatment retention for patients on BZD maintenance treatment was over twice that compared with patients never on BZD prescription (average 72 months vs. 34 months). Lowered mortality in group of patients prescribed BZD briefly/occasionally.
Best, 2002 [20]	UK	Describe patterns of prescribed diazepam and illicit drug use, and levels of anxiety and depression.	Longitudinal	Changes in illicit drug use.	100	30.0% female. Mean age 36.0 yrs (range 22–52 yrs). 96% White.	No change in use of illicit heroin or crack over study period. Prescribed diazepam associated with increased anxiety and depression markers over the 2 years of study; non-prescribed diazepam not associated with changes in anxiety or depressions.
Durand, 2021 [21]	Ireland	Identify determinants of time to dropout from methadone maintenance treatment (MMT) across multiple treatment episodes in specialist addiction services.	Cohort	Time to dropout from MMT at 3 months (90 days) and 12 months (91–365 days).	2035	31.8% female. Median (IQR) age at entry 34.4 (30.2–39.0) yrs.	BZD prescribing (type not specified) was one of several variables studied. Prescribed BZDs were associated with dropout at 12 months as was being male, and number of comorbidities. Low-dose methadone (<60 mg/day) and previous dropout were associated with dropout at 3 and 12 months. Adherence to MMT treatment was protective of dropout at 3 months and 12 months.
Eibl, 2019 [22]	Canada	To assess the impact of prescribed vs. nonprescribed BZD on medication assisted therapy outcomes.	Retrospective cohort study	Retention in treatment.	3692	44.1% female. Mean (SD) age 34.9 (10.03) yrs.	Prescribed BZD (type not specified) had no impact on retention in treatment, but illicit use was associated with poorer outcomes. Non-prescribed BZD use predictive of poorer retention.
Eiroa-Orosa, 2010 [23]	Germany	To analyse the correlation between BZD use, BZD prescription, and treatment outcome among participants in a heroin-assisted treatment trial.	Case–control study	Primary: health improvement (physical and mental); reduction in illicit drug use. Secondary: BZD use; prescription; self-reported addiction severity; psychopathology (anxiety/phobic anxiety focus).	1015	21.1% female. Mean (SD) age 36.5 (6.72) yrs.	Baseline BZD use correlated with lower retention rates but not with poorer outcome. Type of BZD(s) not specified. Ongoing BZD use correlated with poorer outcomes. Significantly better outcomes were found in the course of phobic anxiety symptomatology for those with regular prescription of BZDs. BZD use at entry and during treatment was associated with greater duration and severity of drug problems.
MacLeod, 2019 [24]	UK	To investigate the hypothesis that prescription of BZD in patients receiving OAT would increase risk of mortality overall, irrespective of any increased treatment duration. All 10 BZDs listed in the British National Formulary included.	Observational	All-cause mortality, drug-related poisoning mortality, and mortality not attributable to drug-related poisoning.	12,118	32.7% female. Mean (SD) age at study exit 38.8 (10.4) yrs.	Concurrent prescription of BZD was associated with: (1) Increased duration of methadone treatment (adjusted mean duration of treatment episode 466 days [95% CI 450 to 483] compared to 286 days [95% CI 275 to 297]). (2) Increased risk of drug-related poisoning (adjusted HR 3.34 [95% CI 2.14 to 5.20], *p* < 0.001), with evidence of a dose–response effect. (3) Significant risk for all cause [HR 1.87, 95% CI 1.55 to 2.25, *p* < 0.001]. Concurrent prescription of z-drugs showed evidence of an association with increased risk of drug-related poisoning (adjusted HR 1.64 [95% CI 1.02 to 2.64], *p* < 0.001). Multiple analyses presented in the paper.
Maremmani, 2014 [25]	Italy	Compare long-term outcomes of treatment-resistant heroin addicts (HA) with and without comorbid BZD severe addiction treated with MMT (and CMT for HA + BZD patients). Patients switched to clonazepam.	Controlled cohort study	Retention in treatment, substance use, clinical improvement, general social adjustment, urinalysis.	77	24.7% female. Age range 20-46 yrs.	No differences in survival-in-treatment rates (0.44 vs. 0.58). HA+BZD patients had better outcome results (lower illness severity, better social adjustment) vs. HA patients without BZD severe addiction. HA+BZD patients needed higher methadone dosage in stabilisation phase.
Mijatović, 2013 [26]	Serbia	Assess the safety of low doses of methadone combined with BZD.	Pilot study	QTc interval; self-reported side effects.	20	25.0% female. Mean (SD) age 32.21 (5.63) yrs.	Statistically significant increase in length of QTc interval. Dose-dependent correlation with BZD but not methadone. BZD type not specified.
Mijatović, 2017 [27]	Serbia	Evaluate role of diazepam in methadone -associated QTc prolongation in patients with opioid use disorder during methadone maintenance treatment.	Observational	QTc interval; serum concentration of methadone, diazepam, and electrolytes.	30	20.0% female. Mean (SD) age 32 (5) yrs.	Statistically significant increase in length of QTc interval at 1 month and 6 months. Statistically significant correlation between concentration of methadone and diazepam.
Park, 2020 [28]	USA	To assess whether (1) BZD prescribing during buprenorphine treatment is associated with increased risks of fatal and non-fatal opioid overdose and all-cause mortality; (2) BZD prescribing during buprenorphine treatment is associated with reduced risk of buprenorphine discontinuation (13 prescribable BZD included).	Retrospective cohort study	Primary outcome: fatal opioid overdose. Secondary outcomes: non-fatal opioid overdose, all-cause mortality, buprenorphine treatment discontinuation.	63,345	37.5% female. Mean (SD) age 38 (11.0) yrs.	Of the 67,088 person-years of observation on buprenorphine, 57,825 person-years (86%) represented exposure to buprenorphine alone, and 9263 person-years (14%) represented exposure to buprenorphine and benzodiazepine. 183 people died of an opioid overdose, there were 693 non-fatal opioid overdoses, and 369 people died from any cause. 31% of fatal opioid overdoses occurred during times when people received BZD during buprenorphine treatment. Compared to periods during which people received buprenorphine alone, periods of concurrent BZD and buprenorphine receipt were associated with an increased risk of opioid-related overdose death [HR 2.92, 95% CI 2.10–4.06]. BZD treatment during buprenorphine treatment was also associated with an increased risk of non-fatal opioid overdose (HR 2.05, 95% CI 1.68–2.50) and all-cause mortality (HR 1.90, 95% CI 1.48–2.44). BZD treatment during buprenorphine treatment was associated with a decreased risk of buprenorphine treatment discontinuation (HR 0.87, 95% CI 0.85–0.89). Opioid overdose during buprenorphine treatment was relatively rare: there were only 183 fatal opioid overdoses during the 4-year study period, representing fewer than 4% of the 4754 estimated total opioid overdoses in the state of Massachusetts.
Rapeli, 2009 [29]	Finland	To examine longitudinal (<9 months) changes in memory function among OST patients with BZD use. BZD type not specified.	Longitudinal	Memory function.	43	39.3% female. Mean (SD) age 28.5 (6.55) yrs.	Significantly worse working memory at T1 and T2, and worse immediate verbal memory at T1 in OST patients vs. normal comparison. Both patient groups reported sig. more subjective memory problems vs. comparison at T1 and T2. OST patients with more memory complaints recalled fewer items at T2 from verbal list learned at T1 than patients with fewer memory complaints. No sig. group by time interactions were found.
Schuman-Olivier, 2013 [30]	USA	To evaluate relationship between BZD misuse history, BZD prescription, and both clinical and safety outcomes during buprenorphine treatment.	Secondary data analysis Retrospective chart review with, quasi-experimental design	Clinical outcomes incl. 12-month treatment retention and urine toxicology for illicit opioids); safety outcomes incl. emergency department visits.	328	40.2% female. Mean (SD) age 36.6 (10.65) yrs. 93.0% White ethnicity.	The 12-month treatment retention rate for the sample (N = 328) was 40% (no association between history of BZD misuse OR prescribing AND retention). Poisson regressions of ED visits during buprenorphine treatment revealed more ED visits among those with a BZD prescription versus those without (*p* < 0.001). BZD type not specified. Odds of an accidental injury-related ED visit during treatment were greater among those with a BZD prescription (OR: 3.7; *p* < 0.01), with an enhanced effect among females (OR: 4.7; *p* < 0.01) Overdose was not associated with BZD misuse history or prescription.
Sharma, 2020 [31]	Canada	Estimate the effect of concurrent BZD use on the risk of hospitalisation/emergency department (ED) visits and deaths among people who use opioids.	Secondary analysis Population-based case cross-over study during 2016–2018	Risk of incident all-cause hospitalisation/ED visits; all-cause mortality.	1.06M	55.0% female. Mean (SD) age 48.7 (18.1) yrs.	Concurrent BZD use (type not specified) occurred in 17% of opioid users (179,805/1,056,773). Overall, concurrent use was associated with higher risk of hospitalisation/ED visit (OR 1.13; *p* < 0.001) and all-cause death (OR 1.90; *p* < 0.001). The estimated risk of hospitalisation/ED visit was highest in those >65 (OR 1.5; *p* < 0.001), using multiple health providers (OR 1.67; *p* < 0.001) and >365 days of opioid use (OR 1.76; *p* < 0.001). Events due to opioid toxicity were also associated with concurrent use (OR 1.8; *p* < 0.001). Opioid dose–response effects among concurrent patients who died were also noted (OR 3.13; *p* < 0.001)
Weizman, 2003 [32]	Israel	To compare two pharmacological modalities, clonazepam detoxification and clonazepam maintenance, for treating long-term BZD dependence in methadone maintenance patients	Open—unblinded clinical study.	Reduction or cessation of BZD use	66	No data	In the clonazepam detoxification group, 9/33 (27.3%) were benzodiazepine-free after 2 months. In the clonazepam maintenance group, 26/33 (78.8%) refrained from using additional BZD over the maintenance dose after 2 months. The same success rate remained over the entire year. Survival analysis showed clonazepam maintenance to be more successful than the clonazepam detoxification. Axis I psychiatric comorbidity was found to be positively related to treatment success in the clonazepam maintenance group, while axis II antisocial personality disorder was found to be negatively related to treatment success in that group. It had no impact on the clonazepam detoxification group. Maintenance strategy with clonazepam is a useful treatment modality for benzodiazepine-dependent methadone maintenance patients. Psychiatric comorbidity may have an important role in choosing the adequate treatment modality.
Xu, 2021 [33]	USA	Evaluate association of BZD (converted to diazepam equivalent dose) and z-drug use with non-fatal drug-related poisonings among buprenorphine-maintained patients.	Observational, case-crossover design	Non-fatal drug-related poisoning.	23,036	49.2% female. Mean (SD) age 30.05 (12.15) yrs.	Buprenorphine treatment days associated with 37% reduction in risk of drug-related poisoning events vs. non-treatment days. BZD and z-drug treatment days associated with 88% increase in risk of poisoning events. 78% and 122% increase in poisonings associated with low- and high-dose BZD and z-drug treatment, respectively. High-dose BZD/z-drug treatment associated with increased poisonings in combination with buprenorphine co-treatment; but lower than risk associated with BZD/z-drug treatment in absence of buprenorphine.

BZD: benzodiazepine/HR: hazard ratio; OR: odds ratio; CI: confidence interval; SD: standard deviation.

Studies included a range of benzodiazepines, however, these were generally not specified. For the large epidemiological studies, these were intentionally inclusive. For example, Park et al. [28] included alprazolam, chlordiazepoxide, clonazepam, clorazepate, diazepam, estazolam, flurazepam, lorazepam, oxazepam, prazepam, quazepam, temazepam, and triazolam.

### 3.3. Outcomes Studied

Clinical outcomes included all-cause mortality, drug-related/overdose deaths, treatment retention, hospital emergency care, side effects, QTc interval, and evidence of ongoing illicit substance use. Several studies reported more than one outcome. Grouped outcomes for ACM, overdose deaths, and retention in treatment are presented in Table 3, Table 4 and Table 5 with the effect sizes and confidence intervals.

#### 3.3.1. All-Cause Mortality (ACM)

Five studies reported all-cause mortality (Table 3). Four studies were based on the analysis of existing large datasets, and generally considered to be of good quality. One smaller study using a different design reported on a case note review and structured data extraction at a single general practice, however, it did cover the full population of people prescribed OAT at that practice (n = 178) [19]. Four of the five studies found an increased risk of ACM associated with benzodiazepine prescribing alongside OAT [18,24,28,31]. Bakker and Streel did not find an increase in ACM [19].

**Table 3:** All-cause mortality outcomes.

First Author, Year, Country	Population Studied	Effect Size Estimated for BZD with OAT	95% Confidence Interval	Other Outcomes
Abrahamsson, 2017, Sweden [18]	OAT population (n = 4501)	Significant increase HR 1.75	1.28–2.39	
Bakker, 2017, UK [19]	GP practice OAT Case note review (n = 278)	ACM lower for brief/occasional prescribing compared to BZD maintenance or no BZD		
MacLeod, 2019, UK [24]	Primary care OAT, England (n = 12,118)	Significant increase HR 1.87	1.55–2.25	
Park, 2020, USA [28]	Buprenorphine OAT (n = 63,345)	Significant increase HR 1.9	1.48–2.44	Overdose (HR 1.9)
Sharma, 2020, Canada [31]	Population opioid prescriptions (n = 179,805)	Significant increase OR 1.9 Males OR 2.09 Female OR 1.73	1.76–2.05 1.87–2.33 1.56–1.92	

BZD = benzodiazepine; HR: hazard ratio.

#### 3.3.2. Overdose Deaths (Drug-Related/Drug Poisoning)

Three studies covered overdose deaths (Table 4) [18,24,28]. All were sizeable epidemiological cohort studies. The two largest studies, from the UK and USA, found an increased risk of opioid-related death. The smaller Swedish study reported two analyses informed by different assumptions of the duration of treatment. The shorter treatment time use had a significantly increased hazard ratio compared to the longer time of 90 days in the main analysis, which was not significant [18]. Macleod et al. found a high increased risk of drug-related poisoning for concurrent prescribing (HR 2.96 [95% CI 1.97 to 4.43) [26].

**Table 4:** Overdose deaths.

First Author, Year, Country	Population Studied	Effect Size	95% Confidence Interval	Other Outcomes
Abrahamsson, 2017, Sweden [18]	OAT population (n = 4501)	Not significant HR 1.4, (90 day exposure)	0.97–2.29	Increased non-overdose cause of death (HR 2.02, 95% CI 1.29–3.18). Sensitivity analysis of 30 days exposure was significant.
MacLeod, 2019, UK [24]	Primary care OAT, England (n = 12,118)	Increased HR 3.34	2.14–5.20	Multiple other analyses. Retention increased.
Park, 2020, USA [28]	Buprenorphine OAT (n = 63,345)	Increased HR 2.92	2.10–4.06	Retention increased.

#### 3.3.3. Retention in Treatment (Treatment Duration)

Seven studies measured the treatment retention (Table 5) and included a heterogeneous range of study designs and analyses that were not directly comparable. Benzodiazepine prescribing either improved the retention in treatment [19,24,28] or had no measurable effect [25,30]. An Irish naturalistic study of treatment clinics reported variable findings at different time points with no impact at three months but increased risk of dropout if a person received a benzodiazepine prescription in the previous 90 days [21]. In this study, benzodiazepine prescribing was one of several variables under investigation. Macleod et al. looked at retention by OAT drug with a benzodiazepine and found a higher retention with methadone compared to buprenorphine [24]; however, this did not account for potential confounders as those on methadone and buprenorphine may not be comparable.

**Table 5:** Retention in treatment.

First Author, Year, Country	Population Studied	Retention in Treatment Outcome
Bakker, 2017, UK [19]	GP practice OAT case note review (n = 278)	Treatment retention for patients on BZD maintenance treatment was over twice as long as that compared with patients never on BZD prescription (mean of 72 months vs. 34 months between groups).
Durand, 2021, Ireland [21]	Specialist treatment clinics (n = 2035)	BZD in previous 90 days increased treatment dropout rates at 12 months (HR 1.22, 95% CI 1.03–1.45) but not at 3 months (HR 1.03, 95% CI 0.88–1.21).
Eibl, 2019, Canada [22]	Clinic population (n = 3692)	1-year treatment retention non-prescribed BZD users were two times as likely (adjusted OR 0.38, 95% CI 0.27–0.53) to discontinue treatment as those not using BZD or those using prescribed BZD.
MacLeod, 2019, UK [24]	Primary care OAT, England (n = 12,118)	Concurrent prescription of BZD was associated with increased duration of methadone treatment (adjusted mean duration of treatment episode 466 days [95% CI 450 to 483] compared to 286 days [95% CI 275 to 297]) and for buprenorphine, 234 [95% CI 217 to 250]).
Maremmani, 2014, Italy [25]	(n = 77)	No differences in survival-in-treatment rates (0.44 vs. 0.58).
Park, 2020, USA [28]	Buprenorphine OAT (n = 63,345)	BZD treatment during buprenorphine treatment was associated with a decreased risk of buprenorphine treatment discontinuation (HR 0.87, 95% CI 0.85–0.89).
Schuman-Olivier, 2013, USA [30]	(n = 328)	No statistically significant differences in 12-month retention in treatment based on past-year BZD use BZD Rx or the combination.

A Canadian team studied the impact of prescribed versus street benzodiazepine use among OAT patients receiving methadone. Prescribed benzodiazepines improved methadone maintenance treatment retention whereas non-prescribed benzodiazepines were predictive of treatment dropout [22]. This study is particularly important because it explicitly considered prescribed versus street benzodiazepines, thus acknowledging that there may be different associated risks.

#### 3.3.4. Hospital Emergency Encounters/Hospitalisation

Two studies reported outcomes on hospital emergency encounters. Schuman-Olivier et al. found that the odds of an accident or injury-related emergency department (ED) visit were higher in those with a current benzodiazepine prescription (OR: 3.7, CI 1.81–7.75 *p* < 0.01), with a greater effect in females (OR: 4.7, *p* < 0.01). For other medical causes excluding overdose, there was still an enhanced risk (OR 2.09, CI: 1.15–3.82, *p* < 0.05) [30].

Sharma et al. analysed the risk of hospitalisation/ED visit specifically due to opioid toxicity and found that this increased in those on benzodiazepines (OR 1.13, CI 1.10–1.17, *p* < 0.001). It was highest in those aged >65 (OR 1.5; CI 1.39–1.61, *p* < 0.001), accessing multiple prescribers (more than five) (OR 1.67; CI 1.57–1.77 *p* < 0.001), and >365 days of opioid use (OR 1.76; CI 1.66–1.86, *p* < 0.001) [31]. Considering the total days of concurrent use prior to hospitalisation/ED, one of the highest risks was observed in those who had concurrent use of less than a month (1–30 days) (1.4% vs. 5.8%; OR 2.47; *p* < 0.001).

#### 3.3.5. Other Outcomes

A number of studies that covered other clinical outcomes were included in this review as they could be relevant to prescribing practice in the absence of RCT evidence. The impact of benzodiazepine prescribing on QTc (a periodic measure of heart function, which if prolonged, can be fatal) were studied in two small clinical studies in Serbia. The methods were at times unclear in these studies [26,27]. In the first study, multiple linear regression of factors associated with QTc reported a significant dose-dependent correlation between diazepam dose alongside methadone and QTc (R^2^ = 0.47, *p* = 0.008), but no significant dose-related effect for methadone alone [26]. The second study found a statistically significant increase in length of QTc interval, which correlated with methadone dose in males but was below the risk threshold. This remained a statistically significant correlation when the diazepam dose was included. Baseline QTc: 407.54 ± 27.90; 1 month: 423.17 ± 18.18 (R^2^ = 0.347, *p* = 0.026); and 6 month: 421.39 ± 12.73, (R^2^ = 0.513, *p* = 0.009). Although the authors concluded that concurrent benzodiazepine and OAT use may be a factor in QTc prolongation, these studies were of insufficient quality for a stronger conclusion [27].

Only one study specifically assessed cognitive function [29]. However, the comparison was with those on OAT and a benzodiazepine with a non-drug using comparison group (rather than OAT without a benzodiazepine). They found significantly worse working memory at T1 (within 2 months of start of OAT with a benzodiazepine) and T2 (6–9 months), and worse immediate verbal memory at T1 in OAT patients compared to the non-drug using comparison group. Both patient groups reported significantly more memory complaints compared to the comparison at T1 and T2. OAT patients with more memory complaints recalled fewer items at T2 from the verbal list learned at T1 than patients with fewer memory complaints. This implies that working memory may be affected for some time in OAT patients with benzodiazepine use. The high number of memory complaints among OAT patients with benzodiazepine use could indicate memory consolidation impairment [29].

One study compared clonazepam detoxification with maintenance for OAT patients. In the clonazepam detoxification group, 9/33 (27.3%), were benzodiazepine-free after two months. In the clonazepam maintenance group, 26/33 (78.8%) refrained from using additional benzodiazepines over the maintenance dose after two months, and this was maintained over a year of follow up. Survival analysis found clonazepam maintenance to be superior to clonazepam detoxification. Axis I psychiatric comorbidity was found to be positively related to treatment success in the clonazepam maintenance group while axis II antisocial personality disorder was found to be negatively related to treatment success in that group. There was no impact on the clonazepam detoxification group. The authors concluded that a maintenance approach with clonazepam is a useful treatment modality for benzodiazepine-dependent methadone maintenance patients [32].

Best et al. followed a methadone treatment cohort of 100 people for two years. They measured changes in mental health and illicit cocaine and heroin use, a third of which were prescribed benzodiazepines. This study did not rate well on quality assessment and could have been subject to sampling bias, as the recruitment details were not provided. In light of poor quality assessment, it is hard to draw meaningful conclusions from this study other than to highlight that people using benzodiazepines at entry are more likely to have mental health comorbidities and therefore represent a more clinically challenging group [20].

## 4. Discussion

This systematic review identified a small but developing body of evidence across a range of countries with some notably methodologically strong papers in 2020 and 2021 [28,31,33]. Although there was insufficient homogeneity of methods across studies to undertake a meta-analysis, the literature now provides a good background understanding of the risks of benzodiazepine prescribing alongside OAT. Even with the epidemiological cohort studies, there was variation in the analyses, for example in the period of time of concurrent prescriptions. Macleod et al. undertook multiple analyses of concurrent and co-prescribing that covered periods off treatment. There were also some minor differences in defining overdose death or drug-related poisoning. At times, the terminology differed but the concepts were broadly the same. A pragmatic and inclusive approach was adopted in which a death attributed to drug use was used. Some studies covered methadone, others buprenorphine, and a few covered both medications. There was an indication of differences by OAT drug (e.g., Macleod et al. found better retention with methadone and a benzodiazepine compared to buprenorphine) [24]. A further analysis of Scottish data published since this review found no significant differences by drug with no increase in ACM with buprenorphine (HR 1.16, *p* = 0.189, CI: 0.93–1.44) but an increase with methadone (HR 1.41, *p* < 0.001, CI:1.32–1.50) [34]. Further analysis should undertake comparative analysis by OAT type whilst being mindful of sample bias for non-controlled trials.

All-cause mortality risk was elevated (range 75–90%) in all but one study reporting this outcome. The magnitude of the increased risk across these studies was in a similar range. It is therefore reasonable to conclude that concurrent prescribing of a benzodiazepine alongside OAT increases ACM. Interestingly, a Scottish study published since found a lower risk (17% risk, HR 1.17, CI 1.10–1.24); the adjusted hazard ratio for ACM was 1.17 (1.10 to 1.24)), albeit still significant [34]. Fewer studies have examined overdose specific deaths, and two out of three reported increased risk of drug-related mortality, HR 2.92 (CI 2.10–4.06) and HR 3.34 (CI 2.14–5.20). For non-drug-related deaths, the cause of death is of interest, as concurrent use of two sedative drugs will increase the risk of accidents and falls. However, there is little detail on the causes of death other than drug poisoning in the literature.

Confounding factors may exist such as higher psychiatric comorbidity in the benzodiazepine using/prescribed groups. Indeed, Brands et al. noted the different clinical profiles in people who used benzodiazepines, highlighting there were more women and more psychiatric conditions, providing evidence that combined benzodiazepine and opioid users have more comorbid risk [35]. Epidemiological studies of administrative datasets are subject to bias, and there were no RCTs that covered the main outcomes of mortality, overdose, or retention in treatment. A future RCT is recommended to eliminate confounding factors such as certain comorbidities.

The evidence on retention in treatment is indicative of a positive effect overall, with increased retention in those prescribed a benzodiazepine compared to those not prescribed or those taking non-prescribed benzodiazepine. Similarly, a recently published Scottish study reported longer retention for those co-prescribed a benzodiazepine with OAT (541 days longer, 95% CI 528.56 to 552.61 days) compared to OAT alone. Overall improved retention in treatment is an important clinical consideration. Increased retention allows for more time to engage in psychosocial interventions to address multiple issues including mental health comorbidities that may contribute to street benzodiazepine use. OAT is protective of drug-related death [36] and improved mortality generally [37].

There are other clinical risks of note in concurrent use. Emergency department encounters increased with concurrent prescribing, this being higher in women [30] and older people using benzodiazepines and opioids [31]. Falls and accidents may be part of the explanation as well as overdose risk. Psychiatric comorbidity also has an important role in considering treatment options and influencing treatment outcome. Furthermore, it may be that people are self-medicating with illicit benzodiazepines to manage their mental health conditions, which needs a more detailed assessment and possibly an alternative treatment. Regarding QT interval and the co-consumption of opioids and benzodiazepines, the evidence was inconclusive [27].

Only one study explored memory impairment in depth and found a high number of memory complaints among OAT patients with benzodiazepine use, which could indicate memory impairment [29]. This has considerable implications for the safety of people using benzodiazepines with OAT, if the harm reduction and medication safety messages are not retained. Addition resources to support harm reduction information may be required. Further study is recommended to understand the implications for patients of impaired memory and cognitive impairment. Further studies are also urgently required to assess the reversibility or permanence of memory changes in people taking long-term benzodiazepine, often at high doses.

These studies have limitations in scope and methods. There is little evidence in the literature of the effect of the doses of benzodiazepine, apart from small randomised controlled trials by Lintzeris that indicated a dose-related performance deficit [38,39]. This is important as both effect sizes and side effects are likely to be dose related. These requires further analysis in future studies. Furthermore, the consideration of the equivalence of effect between a pharmaceutically prepared product and an illegally manufactured atypical benzodiazepine is opaque. If neuronal damage or cognitive loss is irreversible, then the cumulative effect over time is likely to be a critical factor. This may impact the effectiveness of OAT, but the nature of impact is unknown.

A methodological weakness across this body of literature is that studies were inclusive of a range of benzodiazepines and did not generally differentiate between benzodiazepines, with the exception of Xu et al. [33]. Furthermore, there was variation in defining the prescription length and the prescribing intention (i.e., short-term, maintenance, slow, or extended detoxification). However, it is recognised that the prescribing/clinical intention can change over time. A randomised controlled trial would ensure equivalence across these variables.

## 5. Conclusions

Clinicians need to weigh up the risks to patients of exposure to unregulated benzodiazepines against the impact of a prescribed alternative (recognising that unregulated benzodiazepines will still be available). The current guidelines for prescribers understandably take the view that benzodiazepines are inherently likely to increase the risk of overdose and death, and therefore to avoid the danger of being implicated in causing harm. However, the protective effect of increased retention in treatment should be considered, as prescribing a benzodiazepine could potentially draw vulnerable people into supportive services. If this were to be pursued, the importance of psychosocial interventions must be considered, given the evidence that people using benzodiazepine may be more prone to mental health conditions. This may require a redesign of services to provide intensive support and treatment monitoring.

In conclusion, there is a growing body of evidence from methodologically robust epidemiological studies, however, there were no randomised controlled trials covering ACM, overdose, or treatment retention. Such randomised controlled studies will take time to provide the evidence required. In the meantime, clinicians should draw on the evidence presented here to weigh up the relative risks of increased ACM versus increased retention in treatment and the risk of continued exposure to an unregulated street market.

## Data Availability

Search and review data can be made available via the lead author.

## Supplementary Materials

The following supporting information can be downloaded at: https://www.mdpi.com/article/10.3390/pharmacy12050152/s1, Supplementary File S1: Search terms.
