# Supplementary material for: Clinical Outcomes of Benzodiazepine Prescribing for People Receiving Opioid Agonist Treatment: A Systematic Review of the Evidence
Source: Pharmacy (Basel). 2024 Oct 4;12(5):152. doi: 10.3390/pharmacy12050152 (PMC11511121; doi:10.3390/pharmacy12050152)
Supplement: Supplementary file 1 [file pharmacy-12-00152-s001.zip › pharmacy-3183432-supplementary.pdf]

pharmacy

Supplementary File S1: Search terms

The following search strategy has been developed to interrogate the Medline database via PubMed. This will be adapted for CINAHL, Embase and Psychinfo.

Search number	Query
1	Alprazolam[Text Word] OR Anthramycin[Text Word] OR Bromazepam[Text Word] OR Chlordiazepoxide[Text Word] OR Clobazam[Text Word] OR Clonazepam[Text Word] OR Clorazepate Dipotassium[Text Word] OR Devazepide[Text Word] OR Diazepam[Text Word] OR Estazolam[Text Word] OR Flunitrazepam[Text Word] OR Flurazepam[Text Word] OR Lorazepam[Text Word] OR Medazepam[Text Word] OR Midazolam[Text Word] OR Nitrazepam[Text Word] OR Oxazepam[Text Word] OR Prazepam[Text Word] OR Temazepam[Text Word] OR Triazolam[Text Word] OR benzodiazepin*[Text Word]
2	Benzodiazepines[Mesh]
3	eszopiclone[Text Word] OR zaleplon[Text Word] OR "Z-drug"[Text Word] OR "z drug"[Text Word] OR zolpidem[Text Word] OR zopiclone[Text Word]
4	gabapentinoid[Text Word] OR gabapentin[Text Word] OR pregabalin[Text Word]
5	buprenorphine[Text Word] OR methadone[Text Word] OR "opiate replacement"[Text Word] OR "opiate substitution"[Text Word] OR "opioid replacement"[Text Word] OR "opioid substitution"[Text Word] OR "opioid maintenance"[Text Word]
6	Opiate Substitution Treatment[Mesh]
7	#1 OR #2 OR #3 OR #4
8	#5 OR #6
9	#7 AND #8
10	("1991/01/01"[Date - Publication] : "3000"[Date - Publication])
11	#9 AND #10
